# Insights from Zebrafish and Mouse Models on the Activity and Safety of Ar-Turmerone as a Potential Drug Candidate for the Treatment of Epilepsy

**DOI:** 10.1371/journal.pone.0081634

**Published:** 2013-12-13

**Authors:** Adriana Monserrath Orellana-Paucar, Tatiana Afrikanova, Joice Thomas, Yelaman K. Aibuldinov, Wim Dehaen, Peter A. M. de Witte, Camila V. Esguerra

**Affiliations:** 1 Laboratory for Molecular Biodiscovery, Department of Pharmaceutical and Pharmacological Sciences, University of Leuven, Leuven, Belgium; 2 Facultad de Ciencias Químicas, Escuela de Bioquímica y Farmacia, Universidad de Cuenca, Cuenca, Ecuador; 3 Laboratory for Molecular Design and Synthesis, Department of Chemistry, University of Leuven, Leuven, Belgium; Tulane University Medical School, United States of America

## Abstract

In a previous study, we uncovered the anticonvulsant properties of turmeric oil and its sesquiterpenoids (ar-turmerone, α-, β-turmerone and α-atlantone) in both zebrafish and mouse models of chemically-induced seizures using pentylenetetrazole (PTZ). In this follow-up study, we aimed at evaluating the anticonvulsant activity of ar-turmerone further. A more in-depth anticonvulsant evaluation of ar-turmerone was therefore carried out in the i.v. PTZ and 6-Hz mouse models. The potential toxic effects of ar-turmerone were evaluated using the beam walking test to assess mouse motor function and balance. In addition, determination of the concentration-time profile of ar-turmerone was carried out for a more extended evaluation of its bioavailability in the mouse brain. Ar-turmerone displayed anticonvulsant properties in both acute seizure models in mice and modulated the expression patterns of two seizure-related genes (*c-fos* and brain-derived neurotrophic factor [bdnf]) in zebrafish. Importantly, no effects on motor function and balance were observed in mice after treatment with ar-turmerone even after administering a dose 500-fold higher than the effective dose in the 6-Hz model. In addition, quantification of its concentration in mouse brains revealed rapid absorption after i.p. administration, capacity to cross the BBB and long-term brain residence. Hence, our results provide additional information on the anticonvulsant properties of ar-turmerone and support further evaluation towards elucidating its mechanism of action, bioavailability, toxicity and potential clinical application.

## Introduction

Epilepsy is a common neurological disorder characterized by spontaneous recurrent focal or generalized seizures that affect the central nervous system in diverse ways. To date, epilepsy affects about 50 million people worldwide [1] [2]. Although half of all known epilepsy cases can be associated with different etiologies, for the other half there is no identifiable cause. The most frequent causes of epileptic seizures in infants are genetic factors, perinatal hypoxia/asfixia, intracranial trauma, congenital malformations of the brain, or infections [3] [4] [5]. In young children and adolescents, epilepsy is usually related to genetic factors [6], whereas in adults, the etiology can be attributed more to cerebrovascular diseases, head injury and brain tumors [7] [8]. 

The pharmacological treatment of epilepsy starts with the administration of a single antiepileptic drug (AED) suitable for the specific epilepsy syndrome. Selection of the appropriate AED is based on its efficacy, tolerability and safety according to the type of seizure. About 70% of patients become seizure-free with single AED therapy. Nevertheless, around 30% of patients with epilepsy remain resistant to both mono- and combinatorial AED therapy [9] [10] [11]. Combinatorial therapy of AEDs is not the first option as it may potentiate the occurrence of adverse side effects due to neurotoxicity and/or hepato-toxicity [10] [12] [13] [14] [15]. However, it is important to note that side effects have not only been reported for AED combinatorial therapy but also for monotherapy [10] [11] [13] [16]. Hence, the current aim of antiepileptic drug discovery is to identify novel active compounds that can provide a safer option with higher efficacy to control pharmaco-resistant seizures compared to currently available AEDs. Such potential drug candidates may be present in medicinal plants traditionally used against seizures [17] [18] [19] [20], as in the case of *Curcuma longa* [21]. The rhizome powder of *Curcuma longa* (turmeric) is commonly used for food preparation due to its yellow color and characteristic aroma but also in ethnopharmacology because of its reported therapeutic properties. The long-standing role of turmeric in traditional medicine is confirmed by its presence in medicinal preparations described in *Sushruta*’s *Ayurvedic Compendium* (250 B.C.) [22]. Presently, there is growing scientific evidence that supports some of these traditional uses. Most relevant for the field of epilepsy, the anticonvulsant activity of turmeric has been verified in preclinical models. These studies have focused mainly on curcuminoids (curcumin) as active principles [23] [24] [25]. Unfortunately, further clinical development of curcumin as a AED candidate has been hampered due to its poor absorption by the gut and rapid metabolism [26]. Conversely, improved bioavailability of turmeric oil in comparison to curcuminoids has been reported [26] [27] [28]. In addition, our previous report demonstrated that other constituents of turmeric, namely the bisabolene sesquiterpenoids of turmeric oil could effectively delay the onset of chemically-induced seizures in zebrafish and mouse models [21]. 

As a follow-up to our previous work, this research study carried out additional tests on ar-turmerone to assess its anticonvulsant activity and to investigate its probable effects on balance and motor function. Ar-turmerone was selected from the group of active sesquiterpenoids present in turmeric oil due to the chemical stability conferred by its aromatic ring. The capability of ar-turmerone to delay seizure generation was evaluated using two acute rodent models: the i.v. pentylenetetrazol (PTZ) timed infusion test and the 6-Hz psychomotor model of partial epilepsy. These two models were chosen on the basis of their proven competence for identification of AEDs acting through a wide variety of mechanisms of action, including novel targets (e.g. levetiracetam) [29] [30] [31] [32] [33]. Furthermore, neurotoxicity evaluation on potential sedative side effects was completed using the beam walking test [34] [35] [36]. 

Aside from the anticonvulsant activity and neurotoxicity tests, we investigated the concentration-time profile of ar-turmerone in the mouse brain for a more in-depth overview of its bioavailability. Additionally, the effects of ar-turmerone on the expression of seizure-related genes were investigated in zebrafish brains in an effort to identify a rational correlation with the proven anticonvulsant activity of this compound in zebrafish and mouse models. We also quantified changes in the expression levels of the seizure-modulated genes *c-fos* and *bdnf* in zebrafish larval brains [37] [38] [39] [40] [41] [42] [43].

In summary, the present study describes a series of preclinical assays carried out to further investigate the anticonvulsant properties and safety of ar-turmerone as a potential drug candidate for the treatment of epilepsy.

## Materials and Methods

### Chemicals and reagents

Dimethyl sulfoxide (99.9%, spectroscopy grade), poly-ethylene glycol M.W. 200 (PEG200), methanol (HPLC grade), dichloromethane (99%, HPLC grade) and heptane (99%, HPLC grade) were obtained from Acros Organics. Levetiracetam (98%, HPLC grade) and pentylenetetrazol were procured from Sigma-Aldrich. Acetonitrile (HPLC grade) was acquired from Fisher Scientific, sodium valproate from Sanofi-Synthelabo and diethyl ether (HPLC grade) from VWR. Propylparaben and hexane (extra pure grade) were procured from Merck. TRIZOL (ref.15596-026) was obtained from Invitrogen, DNase I (ref.04716728001) from Roche and High Capacity cDNA Reverse Transcription Kit (ref.4368814) from Applied Biosystems. Real-time primers and probes were designed by and purchased from IDT DNA.

### Synthesis of ar-turmerone

Ar-turmerone was synthesized through air oxidation of ketonic sesquiterpenes present in turmeric oil. Thus, commercially available turmeric oil (2.7 g) was heated to 100°C during 48 hours in a 10-ml round bottom flask attached to a reflux condenser. Further isolation of ar-turmerone from turmeric oil was achieved by column chromatography (silica, eluent CH_2_Cl_2_-heptane 90:10).

### Ethics Statement

Procedures for animal experiments described in this study were carried out in accordance with Belgian and European Laws, guidelines and policies for animal experimentation, housing and care (Belgian Royal Decree of April 6^th^, 2010 and European Directive 2010/63/EU on the protection of animals used for scientific purposes of October 20^th^, 2010). This project was approved by the Animal Ethics Committee of the University of Leuven (Ethische Commissie van de KU Leuven, approval numbers P101/2010 and P061/2013).

### Mice (*Mus musculus*)

Male C57BI/6 mice at 8 weeks of age (20–30 g) and male NMRI mice (± 20 g) were acquired from Charles River Laboratories (France). The animals were maintained in a temperature and light controlled quiet room (28 °C; 12/12-hour light/dark cycle) in poly-acrylic cages with pellet diet and water *ad libitum*. Mice were allowed to acclimate for one week (NMRI strain) or two weeks (C57Bl/6 strain; until 10 weeks old). 

NMRI mice were used for the 6-Hz psychomotor seizure assay and quantification of ar-turmerone in brain tissue. For the i.v. PTZ infusion evaluation and the beam walking test, C57Bl/6 mice were used.

### Zebrafish (*D. rerio*)

*AB* adult zebrafish were reared under controlled conditions at a temperature of 28.5°C and a light cycle (14/10-hours light/dark). Eggs were collected from natural breeding and kept in embryo medium (17 mM NaCl, 2 mM KCl, 1.8 mM Ca(NO_3_)_2_, 0.12 mM MgSO_4_, 1.5 mM HEPES buffer pH 7.1–7.3 and 0.6 μM methylene blue) in an incubator under constant light and temperature conditions. Medium refreshment and sorting of embryos/larvae was performed in a daily basis until 7 days post-fertilization (dpf). 

### Evaluation of anticonvulsant activity in the 6-Hz psychomotor seizure mouse model

The protocol for this model was as previously described [44] [45]. NMRI mice (30-35 g) were randomly divided into groups of five animals. A defined period before electrical stimulation (30 min and 24 h in our experimental set-up), vehicle (PEG200:DMSO 1:1) or sample dissolved in vehicle were administered intraperitoneally (i.p.) in a volume of 0.1 ml/kg body weight. Valproic acid (300 mg/kg) and levetiracetam (50 mg/kg) were included in this evaluation as positive controls [33] [46].

Psychomotor seizures are induced by low frequency, long duration corneal stimulation (6 Hz, 0.2 ms rectangular pulse width, 3 s duration) using an ECT Unit 5780 (Ugo Basile, Comerio, Italy). Mice were manually restrained and a drop of ocular anesthetic (0.5% xylocaine) was applied to the corneas before electrical stimuli. The head was placed into the holder of the ECT Unit to achieve corneal contact with the electrodes. Then, electrical current was delivered and the mouse was released immediately in a transparent poly-acrylic cage (32 × 14 × 12.5 cm) for behavioral observation. Following electrical stimulation, control group (vehicle treated) displayed sudden behavioral arrest, whisker trembling, head nodding, facial and mouth jerking, forelimb clonus and dorsiflexion of the tail (*Straub tail*) [33] [44] [46]. Sometimes, stunning behavior was preceded by running automatism [44]. Mice displaying normal exploratory and locomotion behavior within a time period shorter than 45 s after electrical stimulation were considered protected. All experiments were carried out in a blinded manner. 

For each experiment, selection of electrical current was based on its capability to elicit psychomotor seizures in 6 out of 6 control mice [vehicle-treated] starting from 32 - 64 mA [33] [45]. If needed, current was increased until uniform occurrence of convulsions was observed in all animals tested. The ECT Unit is provided with a detector to avoid misplacing electrodes during stimulation. All mice were euthanized at the end of the experiment. 

### Evaluation of anticonvulsant activity in the timed i.v. PTZ infusion model

C57Bl/6 mice were randomly divided into control and treatment groups of five mice each. The methodology for this assay was based on the one described in our previous study [21]. Briefly, mice were restrained for tail infusion and catheterized with 1-cm long, 29-gauge needle attached to a polyethylene catheter. Then, vehicle/sample and PTZ were infused intravenously; vehicle/sample first and after 10 min, PTZ was also administered via catheter (7.5 mg/ml; 150 µL/min). During PTZ infusion, mice were observed for the onset of behavioral events such as ear, tail and myoclonic twitch, forelimb clonus, falling, tonic hindlimb extension and death. PTZ doses needed to trigger these parameters were calculated in reference to time latencies between the start of PTZ infusion and the appearance of the aforementioned convulsive episodes. At death, PTZ infusion was terminated. In case of non-occurrence of death, infusion was stopped at 5 min and surviving mice were euthanized immediately. Control groups were always included in every test due to a variation observed from batch to batch regarding PTZ doses to generate behavioral events in mice. Levetiracetam (50 mg/kg) was also included as positive control [30] [47].

### Beam walking test

C57BI/6 mice (10 weeks old) were divided in groups of five. Blinded evaluation was performed to lessen observer bias. All mice were trained until proficiency to walk along a wooden beam without pausing was achieved. The ‘beam walking test’ apparatus consists of a flat bench (70 x 35 cm) with a wooden beam (70 cm long, 1 cm diameter) located 31.5 cm above the bench and held to it on two posts. Two points on the beam were labeled as ‘start’ and ‘end’ areas to allow timing of the animal when walking across the beam. ‘Start –point’ was the site where the mouse was located to begin the test; the ‘end-point’, the place in front of a dark goal box (20 x 10 x 10.5 cm) with food pellets inside. Following training, every mouse was i.v. administered vehicle (PEG200:DMSO 1:1), sample (ar-turmerone 50 mg/kg) or positive control (diazepam 1 mg/kg) according to the treatment group. Ten minutes after administration, the mouse was placed on the tip of the beam at the ‘start-point’ facing towards the beam. The number of footslips, falls and total time on beam (from ‘start’ to ‘end’ points) were noted [35] [36] [48]. If a mouse fell, the animal was returned to the site where it fell from, until completion of beam crossing [48]. 

### Determination of ar-turmerone in mouse brain extract

#### Ar-turmerone administration

Three NMRI mice per dosage group were analyzed at each time-point. A dose of 50 mg/kg of ar-turmerone in PEG200:DMSO 1:1 was administered intraperitoneally (i.p.). Dose and route of administration were selected on the basis of the effectiveness of ar-turmerone to control seizure generation in the 6-Hz psychomotor seizure test.

#### Collection of samples

Transcardial perfusion was applied to mice after defined time-points of drug administration (15 min, 30 min, 1 h, 6h, 24 h) in order to obtain bloodless brain samples. Brains were separated by dissection, washed with saline solution to remove excess surface blood and dried on filter paper. Brains were individually weighed and mixed with hexane (1.5-fold of weight) using a homogenizer (Polytron® PT1200E, Kinematica). Hexane brain extract (supernatant) was collected after centrifugation at 2000 rpm (10 min). Hexane was selected due to its efficiency in the extraction of lipophilic analytes from brain tissue [49] [50]. 

#### Preparation of samples for HPLC analysis

An aliquot of 100 µL of brain extract was dried by passing a slow stream of nitrogen over the sample at room temperature to eliminate the solvent. Residue was dissolved in 100 µL methanol. Solution was vortexed (1 min) and centrifuged at 10.000 rpm (10 min). For HPLC analysis, an aliquot of 20 µL of the clear supernatant was injected. Internal standard (IS) propylparaben was added to obtain a final concentration of 0.25 µg/mL.

#### RP-HPLC method for determination of ar-turmerone

Brain samples were analyzed on LaChrom *Elite* HPLC System [VWR Hitachi] equipped with diode array detection (DAD). Chromatographic separation was performed on a Phenomenex reversed phase column type Luna 3u C18 [150 × 4,6 mm 3 µm] attached to an Phenomenex guard column C18 (4 × 3 mm). The column was operated at a flow rate of 1 ml/min at room temperature (detection wavelength: 240 nm). Samples were analyzed using gradient elution consisting of HPLC grade acetonitrile and Milli-Q purified water according to Mehrotra et al. [51].

#### Calibration curve

A calibration curve was used to estimate the linearity and reproducibility of the HPLC method. Seven standard solutions with concentrations ranging from 0.5 to 6 µg/mL (n=3) were prepared using methanol. IS was added to the standard solutions to yield a final concentration of 0.25 µg/mL. Samples were analyzed according to described procedures. 

#### Recovery of ar-turmerone

Mouse brain homogenate samples (n=3) were spiked with ar-turmerone to obtain final concentrations of 2 and 4 µg/mL. Likewise, IS was added to all samples (final concentration: 0.25 µg/mL). Extraction and analysis of samples was performed according to aforementioned procedures. 

#### Accuracy and precision (RSD)

Intra- and inter-day accuracy was determined at concentrations of 2 and 4 µg/mL in samples (n=3) that were analyzed three times per day within a 3-day period. 

### Gene expression of brain neuromarkers in zebrafish larvae

Seven-dpf larvae of the *AB* strain (10 per group) were exposed to 46 µM ar-turmerone (AT) or vehicle (1% DMSO in embryo medium) for 1 h with subsequent exposure to 20-mM PTZ. Total RNA from the group of fish was extracted with 1 ml TRIZOL protocol, DNase-treated (DNase I) and subsequently reverse transcribed using random hexamers priming and High Capacity cDNA Reverse Transcription Kit. Synthesized cDNA was quantified using the PrimeTime qPCR assay (Integrated DNA Technologies). Primer and probe sequences are indicated in Table 1. Tubulin was used as a reference gene as the stability of its expression was previously assessed for these treatments against beta-actin (data not shown), whereas the expression levels of tubulin were closer to the genes of interest. 

**Table 1:** Primer and probe sequences for gene expression analysis of neuromarkers in the zebrafish brain.

**Gene**	**forward 5' to 3'**	**reverse 5' to 3'**	**probe 5' to 3'**
***c-fos***	GTATTACCCGCTCAACCAGAC	TCCAGTAACCCTCATTTTGGG	CGCAGCTCAATCCTACAACCCGA
***bdnf***	AGCTGAAGAGACAACTTGCAG	CCATAGTAACGAACAGGATGGTC	TGCGCGGAGGTCTTATCCAAAACA
***gabra1***	ACTCTGCTTTACACCATGAGG	CTCGAGTCCAAACGTACACC	AAATTTGAGAGGGCAAGCATGGGC
***il10***	AGCTCCGTTCTGCATACAAAG	GGTCTCCAAGTAGAAATGCAGG	CAGTCCCTATGGATGTCACGTCATGAAC
***tub***	ACTACACTATTGGCAAGGAGC	GAAACCCTGGAGACCTGTG	TGGTCAGACAGTTTGCGAACCCT

*c-fos*: proto-oncogene *c-fos*, *gabra1*: gamma-aminobutyric acid receptor-A, *bdnf*: brain-derived neurotrophic factor, *il10*: interleukin-10

### Statistical Analysis

All statistical analyses were performed using GraphPad™ Prism v.5 (GraphPad Software, Inc.). Anticonvulsant activity of rodents in the 6-Hz psychomotor seizure mice model was evaluated using Fisher’s exact test. Unpaired Student’s *t*-test was used to determine significant differences among control and treatment groups in the timed i.v. PTZ model. Previous assessment of normality was completed using the D’Agostino & Pearson test. For the beam walking test and gene expression analysis of brain neuromarkers, *p*-values (p<0.05) were calculated using one-way ANOVA followed by Dunnett’s multiple comparison tests. In both cases, the ANOVA factor was ‘treatment group’. ‘*Treatment group*’ refers to treatment with vehicle (PEG200:DMSO), ar-turmerone, or diazepam for the beam walking test and, to vehicle (PEG200:DMSO) and pentylenetetrazol for the gene expression analysis experiments.

## Results

### Synthesis of ar-turmerone

After air oxidation, a yield of 1.4 g (52%) of ar-turmerone was obtained from turmeric oil as a pale yellow liquid; ^1^H NMR (300 MHz, CDCl_3_) δ 7.10 (s, 4H), 6.02 (s, 1H), 3.29 (m, 1H), 2.64 (m, 2H), 2.31 (s, 3H), 2.11 (s, 3H), 1.5 (s, 3H), 1.42 (d, 2J = 7.0 Hz, 3H). No traces of α-, β-turmerone were observed in the ^1^H NMR spectra of the treated oil. The enantiomeric purity of the (R)-ar-turmerone was confirmed by comparison of its optical rotation with the value reported in the literature [52] [53].

### Evaluation of anticonvulsant activity in the 6-Hz psychomotor seizure mouse model

**Figure 1:**
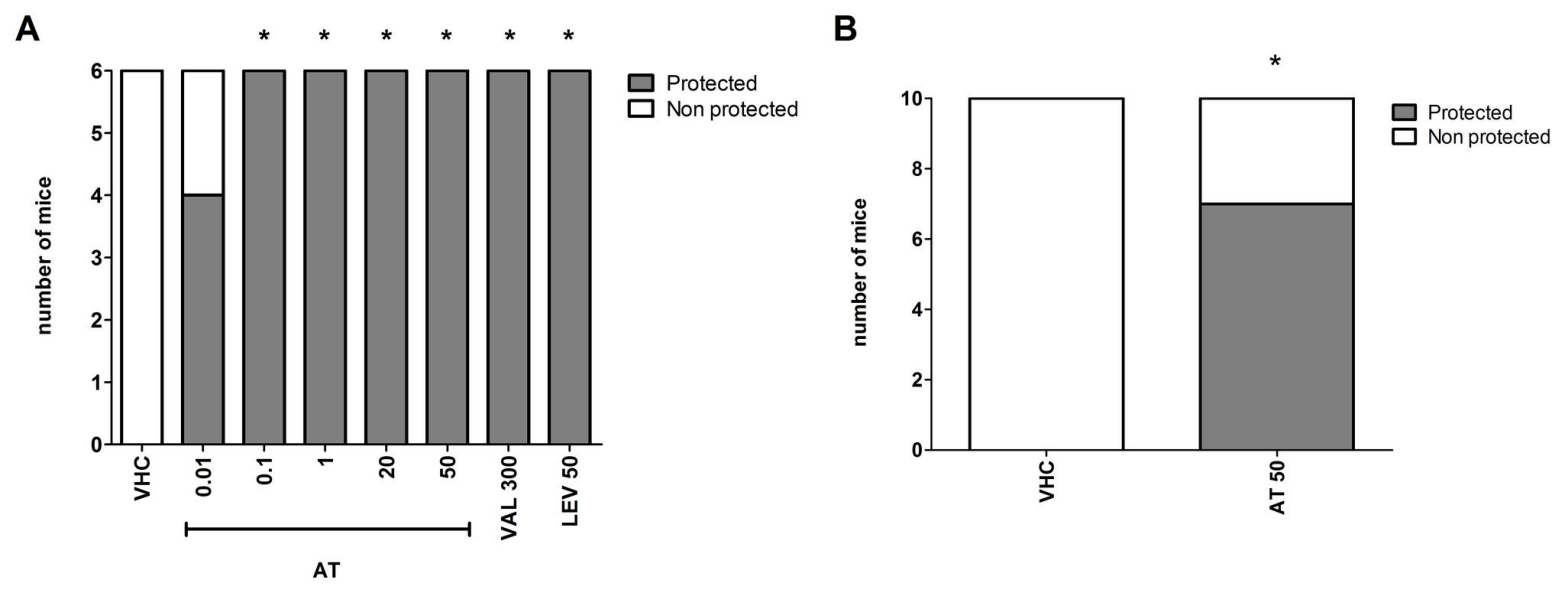
Evaluation of the anticonvulsant activity of ar-turmerone (AT) as determined by the 6-Hz model. A) After 30 min of i.p. administration, complete protection was observed for ar-turmerone at a dose range of 0.1 - 50 mg/kg. A group of vehicle-treated mice (VHC) was included as a negative control. Sodium valproate at 300 mg/kg (VAL300) and levetiracetam at 50 mg/kg (LEV50) were used as positive controls. B) After 24 h i.p. administration (50 mg/kg), protection was observed after electrical induction. Statistically significant differences between control (VHC) and sample groups are labeled as * for p < 0.05 (Fisher’s exact test).

Following electrical stimulation (performed 30 min after ar-turmerone administration), all treated mice (n=6) were protected at doses ranging between 0.1 and 50 mg/kg (p<0.05) (Figure 1A). Even after 24 h i.p. administration of ar-turmerone (50 mg/kg), 70% of treated mice were protected following seizure induction (n=10; p<0.05) (Figure 1B). For positive controls, complete protection (n=6; p<0.05) was detected with levetiracetam (50 mg/kg) and sodium valproate (300 mg/kg) (Figure 1A). Electrical current in all cases was 44 mA.

### Evaluation of anticonvulsant activity in the timed i.v. PTZ -infusion model

**Figure 2:**
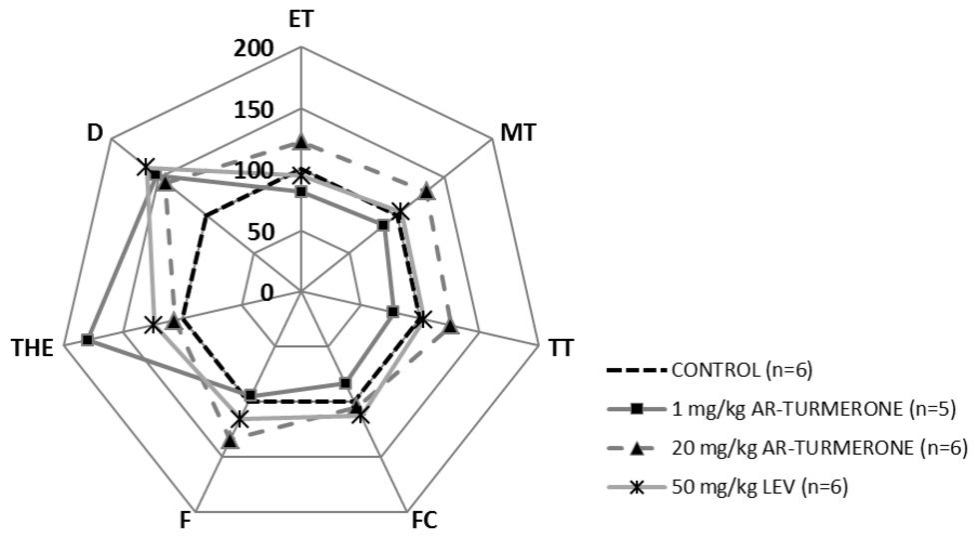
Evaluation of the anticonvulsant activity of ar-turmerone (AT) in the timed i.v. **PTZ infusion model**. Radar graphic depicts the doses of PTZ required to trigger seven characteristic behavioral events (ET: ear twitch, MT: myoclonic twitch, TT: tail twitch, FC: forelimb clonus, F: falling, THE: tonic hindlimb extension, D: death) after i.v. administration of AT at 1 and 20 mg/kg compared to vehicle-treated mice (control). Levetiracetam (LEV) 50 mg/kg was included as a positive control. Results are expressed as relative values compared to control (set as 100%). For reasons of clarity, mean values (± SD) and statistically significant differences are not shown here (for details see Table 2).

PTZ (7.5 mg/ml) infused intravenously at a constant rate (150 µL/min) in control group mice (VHC) elicited characteristic behavioral events: ear, tail and myoclonic twitch, forelimb clonus, falling, tonic hindlimb extension and death. Ar-turmerone at the dose of 1 mg/kg protected mice by raising the threshold of PTZ needed to trigger tonic hindlimb extension and death (p<0.05). Furthermore, ar-turmerone at 20 mg/kg increased the PTZ dose necessary to cause death in mice (p<0.05) (Figure 2; Table 2). A similar increase in the PTZ dose required to ellicit death in treated mice (p < 0.05) was observed for levetiracetam at 50 mg/kg (positive control) (Figure 2; Table 2). 

**Table 2:** Timed i.v. PTZ infusion model: PTZ doses.

	**PTZ dose required [mg/kg]**					
	**Control**	**AT**	**Control**	**AT**	**Control**	**LEV**
	**[VHC]**	**1 mg/kg**	**[VHC]**	**20 mg/kg**	**[VHC]**	**50 mg/kg**
**ET**	73,2 ± 17,4	59,5 ± 19,7	40,2 ± 11,2	49,3 ± 10,7	51,7 ± 11,9	49,0 ± 12,0
**MT**	82,6 ± 20,9	71,4 ± 20,5	44,6 ± 10,3	58,7 ± 15,1	56,3 ± 13,5	56,6 ± 12,8
**TT**	78,7 ± 22,6	61,0 ± 20,5	43,9 ± 12,7	55,1 ± 14,2	53,7 ± 12,1	52,7 ± 13,8
**FC**	93,1 ± 27,2	77,9 ± 24,2	66,4 ± 14,8	69,4 ± 16,0	70,6 ± 16,0	79,5 ± 26,1
**F**	89,5 ± 14,1	85,2 ± 19,1	59,5 ± 21,4	76,6 ± 14,5	66,3 ± 12,7	77,5 ± 25,9
**THE**	111,9 ± 17,5	162,3 ± 43,3 *	110,0 ± 20,1	117,4 ± 26,3	100,6 ± 16,7	119,5 ± 23,2
**D**	134,0 ± 20,7	196,6 ± 42,2 *	132,8 ± 18,8	189,8 ± 55,3 *	120,1 ± 18,9	185,2 ± 44,8 *

ET: ear twitch, MT: myoclonic twitch, TT: tail twitch, FC: forelimb clonus, F: falling, THE: tonic hindlimb extension, D: death

VHC: vehicle, AT: ar-turmerone, LEV: levetiracetam

Values are expressed as mean values (± SD). Statistically significant differences between sample and control group are labeled as * for p < 0.05 (unpaired Student's t-test).

### Beam walking test

**Figure 3:**
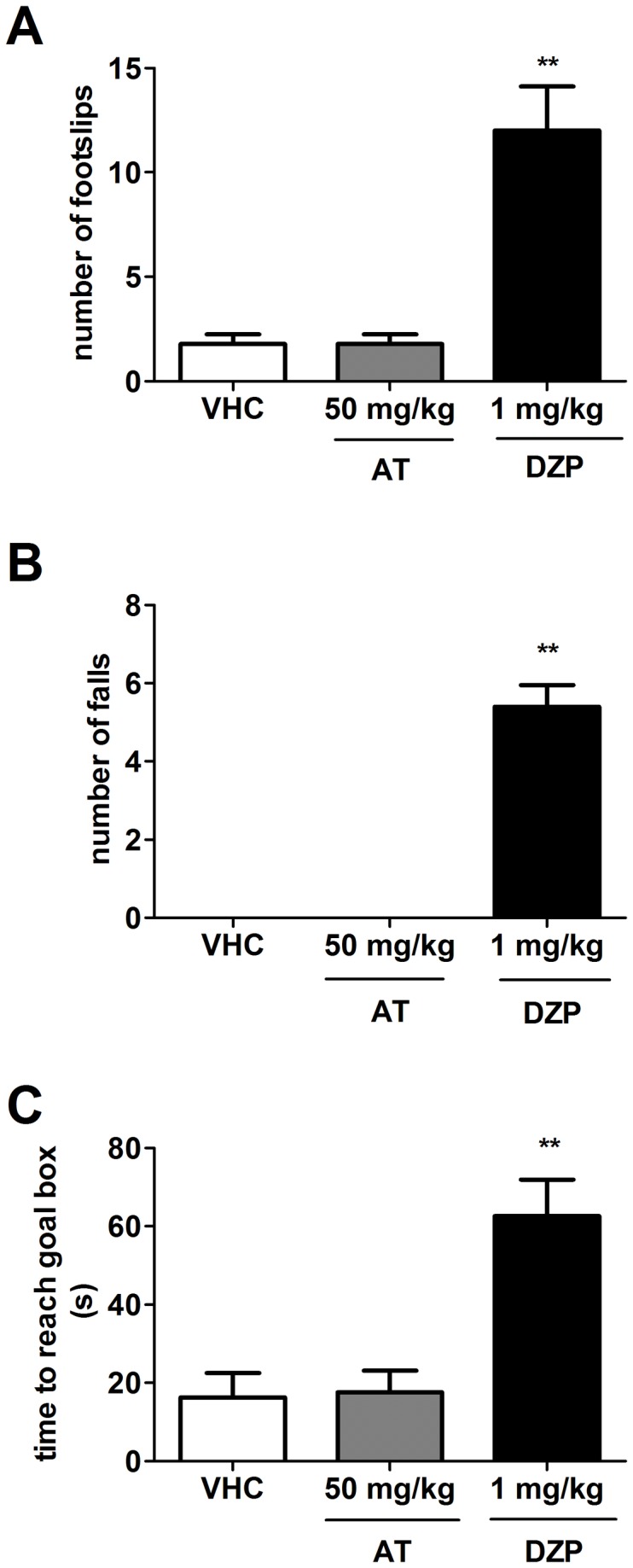
Evaluation of the effect of ar-turmerone on mouse motor function and balance. An intravenous dose of 50 mg/kg of ar-turmerone (AT) does not cause any alteration of motor skills in mice. Sensitivity of this model was confirmed by detection of motor and balance deficits induced by diazepam (DPZ) in mice at 1 mg/kg. Compared to control group (VHC), DZP-treated mice displayed a significant increase in number of footslips (A), falls (B) and time to reach goal box (C). Statistically significant differences between control and sample groups are labeled as ** for p < 0.001 (one-way ANOVA test).

The dose of ar-turmerone and the administration route used in this assay were based on the same conditions that showed anticonvulsant activity in the i.v. PTZ model. No effect on balance was observed in C57Bl/6 mice after i.v. administration of ar-turmerone (50 mg/kg) in this motor function test. Behavior and performance of mice treated with ar-turmerone were comparable to the control group (VHC) (Figure 3). Diazepam (DZP) significantly increased the number of foot slips (p<0.05) and the occurrence of falling (p<0.05) as well as the total time needed for mice to cross the beam (p<0.05) (Figure 3). Likewise for NMRI mice, no differences in motor function/balance were observed between vehicle-treated (control group) and ar-turmerone-treated animals after an intra-peritoneal dose of 50 mg/kg (data not shown).

### Determination of ar-turmerone in mouse brain homogenate

#### Selectivity

**Figure 4:**
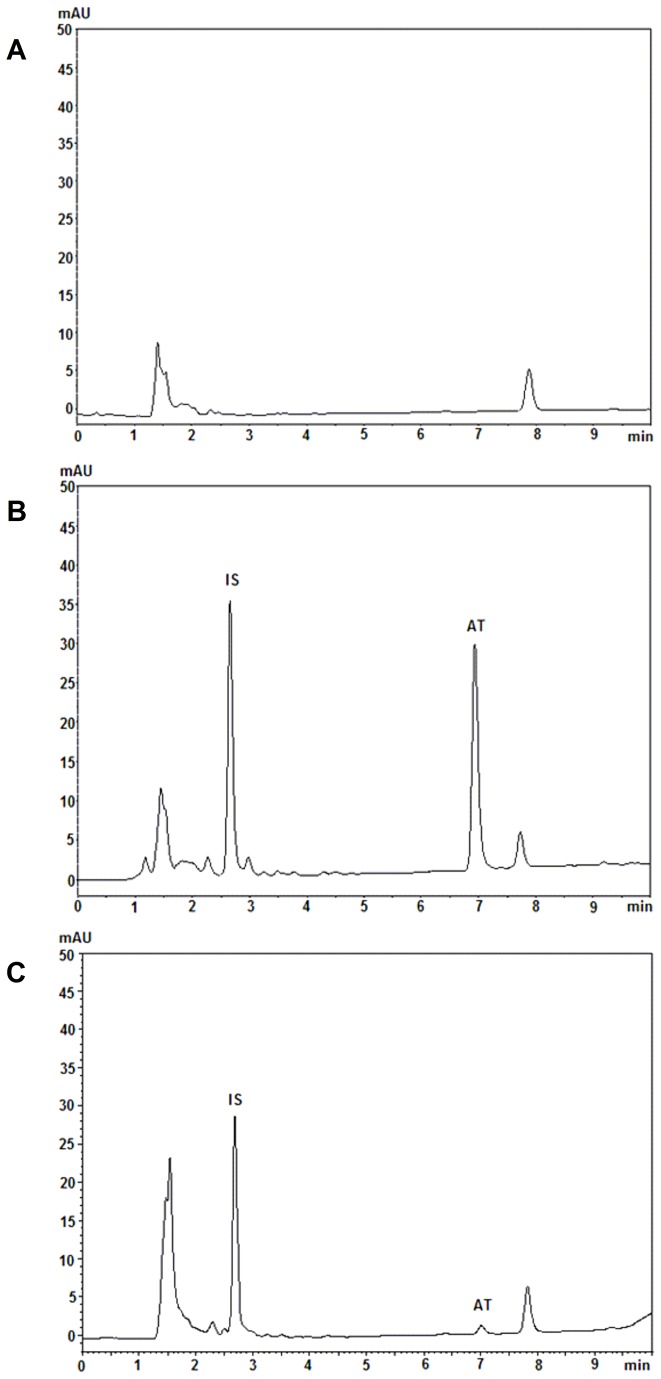
Representative HPLC chromatograms of mouse brain homogenate analysis for ar-turmerone determination. A) blank mouse brain extract, B) blank mouse brain extract spiked with ar-turmerone (AT) and internal standard (IS) and C) brain extract sample of a dosed mouse.

The method for the chromatographic analysis of brain homogenate samples showed no interference from endogenous components at the retention times of IS (2.6 min) and ar-turmerone (6.9 min) in the chromatograms. Therefore, it was found to be specific and efficient for our purposes (Figure 4).

#### Linearity

The calibration curve of ar-turmerone from standard solutions was linear over the concentration range of 0.5 - 6 µg/mL (n=3; r=0.994).

#### Recovery

The mean (± SD) recovery of ar-turmerone from brain homogenate at 2 and 4 µg/mL was 84.2 ± 4.0% and 85.5 ± 10.4%, respectively. 

#### Accuracy and precision

Accuracy and precision of the HPLC methodology for ar-turmerone determination in brain homogenate are listed in Table 3. 

**Table 3:** Intra-, inter-assay accuracy and precision of the method for determination of ar-turmerone in brain extracts.

Concentration [ug/mL]	Accuracy %		RSD %	
	Intra-assay	Inter-assay	Intra-assay	Inter-assay
2	99,7	99,5	1,4	0,1
4	99,1	100,5	2,9	1,2

#### Determination of ar-turmerone

**Figure 5:**
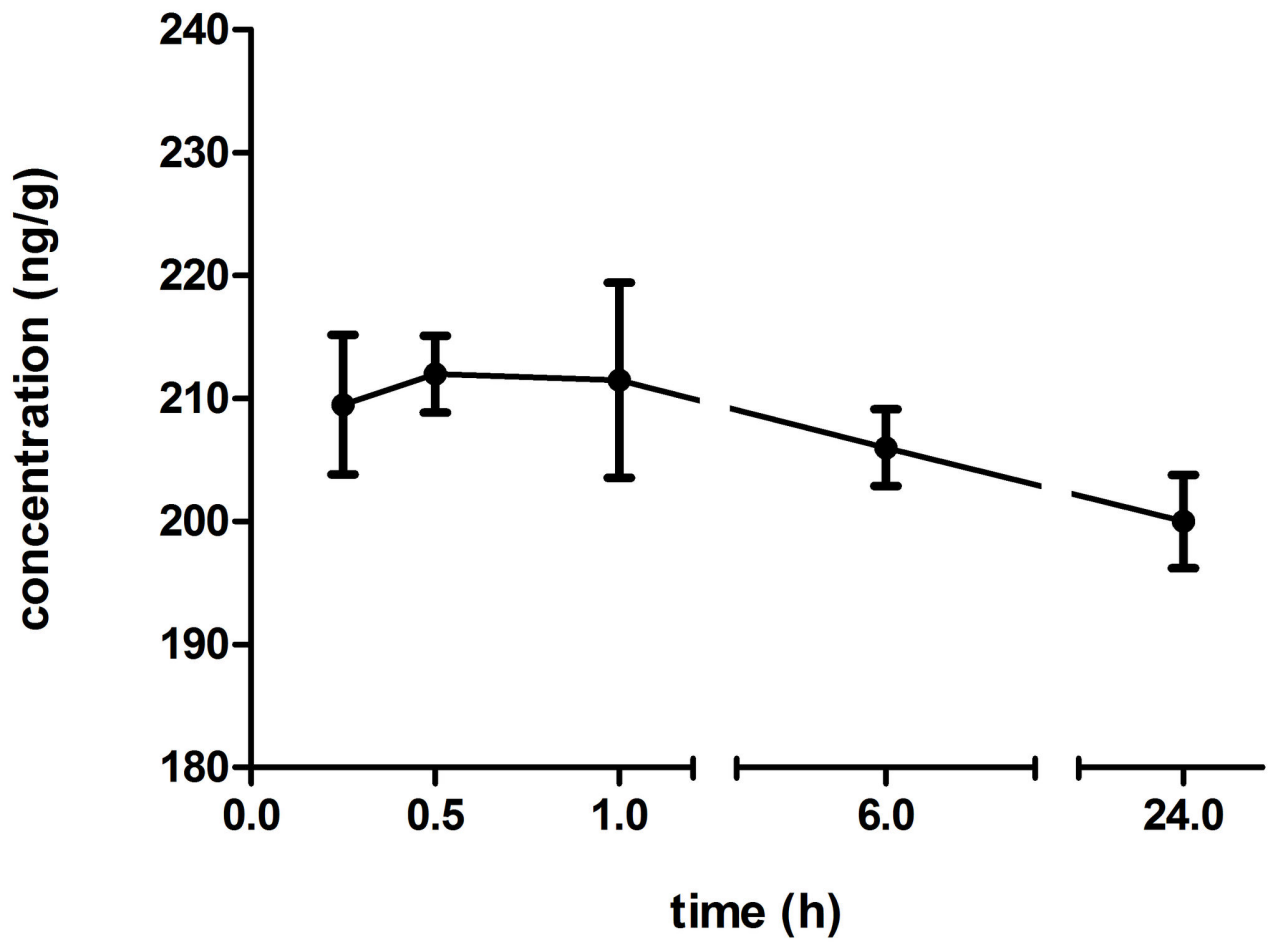
Concentration – time profile of ar-turmerone in mouse brain extracts. Chromatographic analysis of these samples showed evidence of the presence of ar-turmerone at 15 min and up to 24 hours after i.p administration of 50 mg/kg.

The characterized HPLC method was used for determination of the concentration-time profile of ar-turmerone in mouse brain extract. Following i.p. dosage of 50 mg/kg of ar-turmerone, chromatographic analysis of samples showed evidence for the presence of this compound in mouse brain at 15 min and up to 24 hours after administration (Figure 5). 

### Gene expression of brain neuromarkers in zebrafish larvae

**Figure 6:**
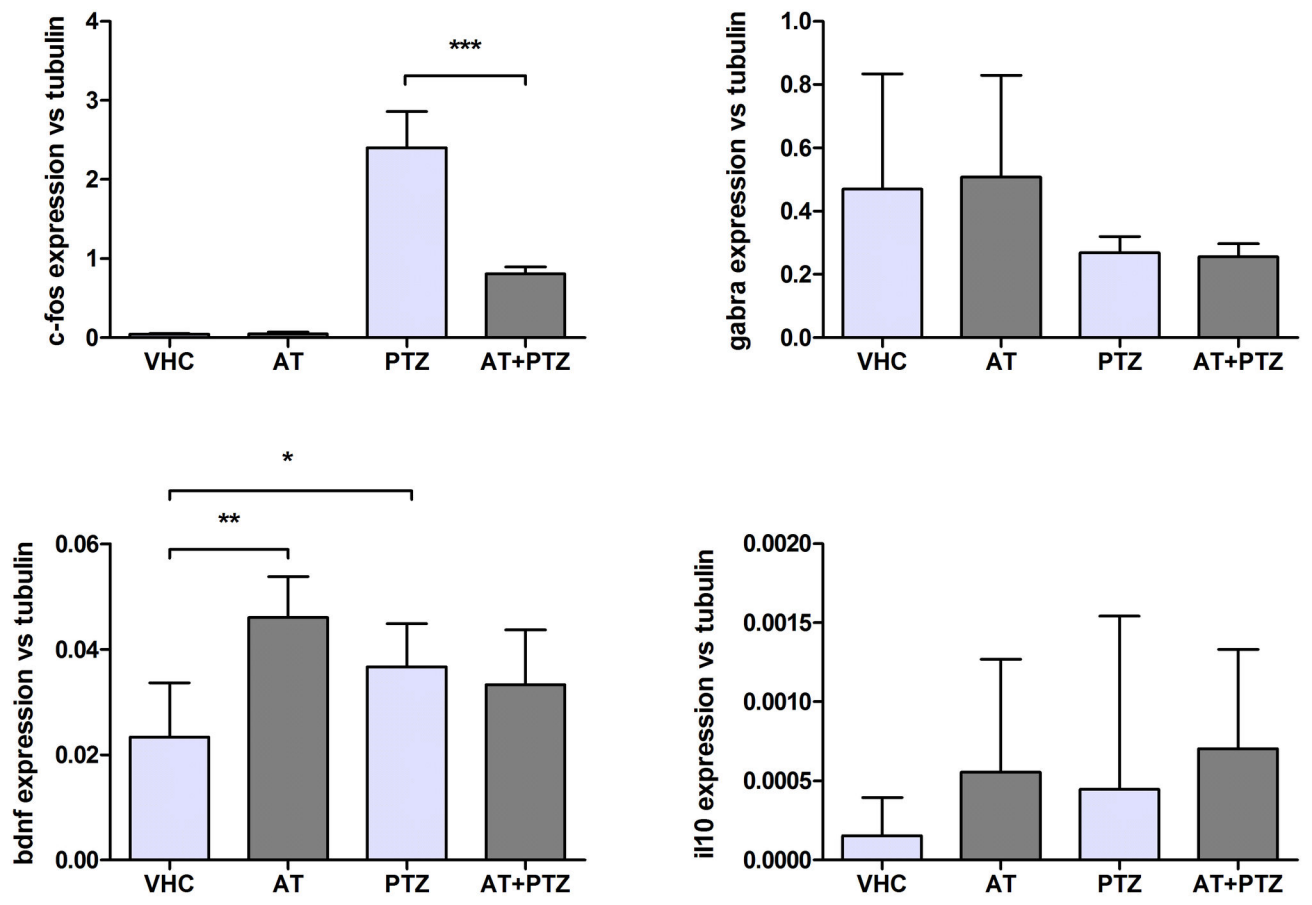
Evaluation of the effect of ar-turmerone on seizure-related marker expression in zebrafish brains. Ar-turmerone (AT) downregulated PTZ-induced *c-fos* expression and upregulated *bdnf* expression in the abscense of PTZ treatment. Statistically significant differences between groups: control (VHC), vehicle treated exposed to PTZ (PTZ), ar-turmerone treated (AT) and ar-turmerone treated exposed to PTZ (AT+PTZ) are labeled as * for p < 0.05 and ** for p < 0.001 (one-way ANOVA test).

Concentration of ar-turmerone used in this test was determined by previous results of positive anticonvulsant activity at 46 µM in the zebrafish model [21]. No signs of toxicity such as lack of startle response to plate taps, changes in heart rate or circulation, presence of edema, loss of posture, paralysis and/or death were observed in zebrafish larvae at this concentration. Ar-turmerone significantly downregulated *c-fos* epression induced by PTZ (p<0.001). In the absence of PTZ, *bdnf* expression was upregulated in the ar-turmerone treated group (p<0.01). After PTZ treatment, *bdnf* was highly expressed in both groups (vehicle-treated and ar-turmerone-treated larvae); increase of *bdnf* expression was found to be significant for the vehicle-treated group when compared to the control group (p<0.05). Expression patterns of the GABA-_A_ receptor and IL10 were not affected by ar-turmerone treatment (Figure 6).

## Discussion

In line with NIH guidelines [54], we conducted our research with the goal of using key acute seizure models that could potentially increase our chances of identifying compound/s acting through novel mechanisms of action or that might constitute a safer alternative than the available pharmacological options. In this study, we also propose a simple and efficient method for synthesis and isolation of ar-turmerone from turmeric oil, as further in vivo characterization of the pharmacological properties of ar-turmerone will require considerably larger amounts of compound. Turmeric oil is composed mainly of bisabolene sesquiterpenoids such as ar-turmerone, α-turmerone, β-turmerone and α-atlantone [55]. It has been reported that ketonic sesquiterpenes of turmeric oil such as α-turmerone and β-turmerone can be converted to ar-turmerone by air oxidation over time [52] [53] [56]. Thus, different oxidation conditions were investigated to increase the content of ar-turmerone in turmeric oil. It was observed that oxidation of turmeric oil with p-chloranil at different temperatures did not lead to a complete conversion of α- and β-turmerone to ar-turmerone. Alternatively, treatment with 2,3-dichloro-5,6-dicyano-1,4-benzoquinone (DDQ) at 120°C for 24 h resulted in a satisfactory increase in ar-turmerone yield from 30% to 48% after column chromatography purification. However, remaining chlorinated products (trace amounts) were also detected with this method. A satisfactory result was finally achieved by using air as the oxidizing agent at 100°C over a period of 48 h. Interestingly, no trace of α and β-turmerone was observed in the crude NMR spectra of the treated oil. Hence, this method was found appropriate due to a significant conversion (52%) of different ketonic sesquiterpenes from turmeric oil to ar-turmerone with a high purity level as well. 

For anticonvulsant activity evaluation, the i.v. PTZ timed infusion test and the 6-Hz psychomotor model of partial epilepsy were selected due to their unique sensitivity for identification of AEDs with a wide range of mechanisms of action [29] [30] [31] [32] [33] [44] [57] including detection of the anticonvulsant properties of levetiracetam that were “overlooked” by MES and s.c. PTZ tests [30] [33] [58]. Levetiracetam is an AED acting predominantly on a different target to those already known – i.e. via specific binding to the synaptic vesicle protein SV2A [59] [60]. Our previous results from the i.v. PTZ test revealed a clear anticonvulsant activity of ar-turmerone at a dose of 50 mg/kg [21]. To complete the dose-response analysis for this test, two additional doses of ar-turmerone were evaluated (1 and 20 mg/kg). In both cases, a considerable increase in PTZ dose required to cause death in mice was observed. Positive activity of levetiracetam in this model confirms previous results from Mandhane et al. [30] and corroborate its sensitivity for identifying potential drug candidates likely acting through novel mechanisms of action. 

In addition, the clear protective activity of ar-turmerone in the 6-Hz model revealed its potency in controlling seizure generation within an extensive range of effective doses (0.1 - 50 mg/kg) at 30 min and up to 24 h after i.p. administration. Along with its demonstrated potency, these results provide evidence for a wide therapeutic window that potentially lowers the chance of side effects. A correlation between the protective activity of levetiracetam in the 6-Hz model [33] [46] [57] [58] [60] [61] and its proven effectiveness as a monotherapy and ‘add-on’ drug for the treatment of refractory human epilepsies [62] [63] [64] [65] [66] supports the usefulness of this acute model for the identification of AED candidates acting through novel mechanisms of action. 

Evaluation of potential toxicity is also crucial in the search for novel AEDs. For this purpose, we used the beam walking test which possesses higher sensitivity when compared to the rotarod test for measuring motor function and balance in mice [48]. Results from the beam walking test lead to a more accurate prediction of benzodiazepine-induced motor coordination deficits at relatively low GABA-_A_ receptor occupancy (e.g 30% for diazepam) in contrast with rotarod performance which is affected only at much higher levels (72%) of receptor occupancy [48] [67]. It has been stated that evaluation of known AEDs in the beam walking test is more closely related to what is reported in clinical trials [67]. After i.v. and i.p. administration of 50 mg/kg of ar-turmerone [highest tested active dose], mice did not display any alteration in beam walking performance. No significant differences in the number of footslips, falls and time on beam were observed when compared to the vehicle injected-group. Consistently, identical results were found in NMRI mice after i.p. administration of 50 mg/kg [data not shown]. These results suggest ar-turmerone as a potential drug candidate with no effect on locomotor activity or balance in rodents and a high probability of comparable safety in humans as well. Our results are consistent with the findings of Liju et al. regarding non-toxicity of turmeric oil, containing 61.7% ar-turmerone, after acute and subchronic oral administration in rats [68]. The safety of the main constituent of turmeric oil, ar-turmerone, has also been verified by studies in humans after oral administration of turmeric oil over three months [69]. With respect to safety, one of the most common side effects of the available AEDs is cognitive impairment. Turmeric exhibits an additional advantage in this regard. Better cognitive performance in elderly people has been associated with turmeric consumption [70]. Of course, whether ar-turmerone is the main constituent of turmeric responsible for this observed increase in cognitive performance remains to be determined. Nevertheless, the potential add-on benefit if this were to be the case is intriguing to say the least. Furthermore, the use of turmeric for centuries as a spice, especially in Indian food, confers additional support for its safety for human consumption. In fact, it has been reported that a diet including daily consumption of turmeric of up to 1.5 g or even 4 g per person (around 10 - 30 mg of ar-turmerone) is not associated with adverse effects in humans [71] [72] [73]. 

Noteworthy is the relationship between the positive activity of ar-turmerone in the i.v. PTZ seizure test and the safety displayed in the beam walking test. The i.v. PTZ test is able to identify a wide variety of AEDs acting through different mechanisms of action including GABA-related AEDs [30]. Seizure generation is then controlled through enhancement of GABAergic transmission in the case of GABA-related AEDs [31] [74]. However, these AEDs are known to induce muscle hypotonia and sedation as side effects [e.g. diazepam] [75] [76]. Thus, the use of the beam walking test is not only constrained to evident impairment of locomotor function/balance after AED administration but also provides important hints about the potential mechanisms of action of ar-turmerone. Normal performance of mice in this test after administration of ar-turmerone suggests that the anticonvulsant activity of ar-turmerone may not be mainly exerted by GABA-mediated inhibition. Confirmation of the anticonvulsant properties of ar-turmerone without sedation constitutes an important advantage over the known AEDs (at least in the preclinical stage). Indeed, more specific studies about the possible mechanism/s of action of ar-turmerone are still needed.

Determination of the concentration - time profile of ar-turmerone in mouse brains after i.p. administration (50 mg/kg), evidenced rapid absorption from the peritoneum to the brain. Detection of ar-turmerone in brain tissue was possible after 15 min and up to 24 hours after administration. This finding is consistent with the results from the 6-Hz model where protection against electrical stimulation is observed in 70% of treated mice up to 24 h after i.p. administration of ar-turmerone. Our results underscore the ability for ar-turmerone to penetrate the BBB and, in line with Prakash et al. [27], supports its high absorption and bioavailability. High potency of ar-turmerone may be supported by the fact that detected concentrations in the brain are quite low if compared to available AEDs. After administration of effective doses of different well-known AEDs, levels in brain tissue were found to be between the range of 2.59 - 26.61 µg/g [77]. On the other hand, detection of low concentrations of ar-turmerone in brain tissue may also be explained by the generation of ar-turmerone metabolites and thus, the true active compound may have been overlooked using this protocol. Certainly, there are additional studies that need to be completed to understand the bioavailability of ar-turmerone better. A further characterization of the pharmacokinetic profile of ar-turmerone is currently in our interest since it will provide additional information about the relationship between the concentration-time profile of the active compound/metabolite in brain and its therapeutic effects, dosage, route and frequency of administration, etc. Interestingly, after achieving peak concentration in brain (212 ng/mg) at 30 min, the levels of ar-turmerone were maintained in the range of 200 ng/ml for up to 24 hours. This finding may be explained by the hypothesis of a ‘depot’ formation of ar-turmerone at level of the peritoneum after administration through this route. Thus, a continued gradual release of ar-turmerone along with its capability to cross BBB could explain its sustained presence in the brain over a long period. 

In addition to the rodent studies, modulation of the seizure-related markers (*c-fos*, gamma-aminobutyric acid receptor-A [gabra], brain-derived neurotrophic factor [bdnf] and interleukin-10 [*il10*]), was analyzed in zebrafish brains after treatment with ar-turmerone in the presence and absence of PTZ. Genes for this evaluation were selected on the basis of their key role in seizure-related pathogenesis. The proto-oncogene *c-fos* is upregulated significantly in the brain in response to triggering stimuli (e.g. seizures) in both zebrafish and rodent models [37] [38] [39] [40]. The gene *gabra* is a crucial link in the main inhibitory neurotransmitter pathway of the CNS, with changes in its expression or mutations are closely associated with epileptogenesis [78]. BDNF upregulation has been associated with antidepressant effects [79] [41] while its deficits have been linked with a number of neurodevelopmental, neurodegenerative, and neuropsychiatric disorders [42]. BDNF also plays a role in the pathogenesis of temporal lobe epilepsy (TLE) by enhancing neuronal outgrowth and mossy fiber sprouting [43]. IL10 prevents neuronal damage caused by excess of excitatory neurotransmitters and reduces the harmful effects of hypoxia as well [80]. Our results show a significant decrease in *c-fos* transcription in larvae treated with ar-turmerone and exposed to PTZ. Thus, considering that *c-fos* is a marker of seizure severity, this result suggests that ar-turmerone plays a crucial role in controlling abnormal neuronal activity accompanying PTZ-induced seizures [37]. In addition, comparison of the results from zebrafish exposed to vehicle only and ar-turmerone only, revealed *bdnf* upregulation in the brain after ar-turmerone treatment. Hence, *bdnf* upregulation may underlie potential antidepressant activity of ar-turmerone and/or neuroprotective effects. On the other hand, an increase of *bdnf* transcription was observed for both groups [vehicle-treated and ar-turmerone treated] after PTZ exposure. This observation is in line with the reported upregulation of BDNF [protein and mRNA] at the hippocampal level in seizure animal models [81][82][83]. Under oxidative stress conditions [e.g. caused by PTZ exposure], an initial marked downregulation of BDNF, triggers a subsequent activation of pro-BDNF pathways [84].. Thus, the increased expression of *bdnf* in the PTZ-only treated group appears to be due to the proconvulsant properties of PTZ. However, the upregulation of *bdnf* expression in the case of ar-turmerone-treated larvae does not appear to be due to an intrinsic proconvulsant activity of the compound as *c-fos* expression is significantly downregulated after combined treatment with ar-turmerone and PTZ. Thus, it could be inferred that the increase in *bdnf* expression in ar-turmerone + PTZ treated larvae was not only ’triggered’ by PTZ but also related to an intrinsic effect of ar-turmerone. Although additional confirmatory studies are clearly warranted, these findings suggest that ar-turmerone may modulate anti-depressant activity mediated by enhancement of BNDF mRNA expression in the brain. 

In summary, our results suggest ar-turmerone as a potentially safe AED candidate with influence on the expression of two relevant seizure-related genes and proven activity in two acute seizure mouse tests (including a proposed pharmaco-resistance model). Future experiments using other models (e.g. kindling, status epilepticus, post-stroke, post-traumatic brain, status epilepticus, genetic models, among others) are still necessary to characterize the potential clinical application of ar-turmerone further. Hence, the present study supports continued research for investigating the anticonvulsant properties of ar-turmerone, likely as an alternative for pharmaco-resistant cases and/or clinical cases of adverse effects with currently available AEDs.
